# Interaction via the N terminus of the type IV secretion system (T4SS) protein VirB6 with VirB10 is required for VirB2 and VirB5 incorporation into T-pili and for T4SS function

**DOI:** 10.1074/jbc.RA118.002751

**Published:** 2018-07-05

**Authors:** Charline Mary, Aurélien Fouillen, Benoit Bessette, Antonio Nanci, Christian Baron

**Affiliations:** From the ‡ Department of Biochemistry and Molecular Medicine, Faculty of Medicine, Université de Montréal, Montréal, Quebec H3C 3J7, Canada and; the § Department of Stomatology, Faculty of Dental Medicine, Université de Montréal, Montréal H3C 3J7, Quebec, Canada

**Keywords:** membrane protein, protein secretion, virulence factor, bacterial pathogenesis, bacteria, Agrobacterium tumefaciens, protein–protein interaction, T-pilus, type IV secretion system, virulence

## Abstract

Many bacterial pathogens employ multicomponent protein complexes such as type IV secretion systems (T4SSs) to transfer virulence factors into host cells. Here we studied the interaction between two essential T4SS components: the very hydrophobic inner membrane protein VirB6, which may be a component of the translocation channel, and VirB10, which links the inner and outer bacterial membranes. To map the interaction site between these two T4SS components, we conducted alanine scanning and deleted six-amino acid stretches from the N-terminal periplasmic domain of VirB6 from *Brucella suis*. Using the bacterial two-hybrid system to analyze the effects of these alterations on the VirB6–VirB10 interaction, we identified the amino acid regions 16–21 and 28–33 and Leu-18 in VirB6 as being required for this interaction. SDS-PAGE coupled with Western blotting of cell lysates and native PAGE of detergent-extracted membrane proteins revealed that the corresponding VirB6 residues in *Agrobacterium tumefaciens* (Phe-20 and amino acids 18–23 and 30–35) modulate the stability of both VirB6 and VirB5. However, the results from immuno-EM and super-resolution microscopy suggested that these regions and residues are not required for membrane association or for polar localization of VirB6. The six-amino acid deletions in the N terminus of VirB6 abolished pilus formation and virulence of *A. tumefaciens*, and the corresponding deletions in the VirB6 homolog TraD from the plasmid pKM101-T4SS abrogated plasmid transfer. Our results indicate that specific residues of the VirB6 N-terminal domain are required for VirB6 stabilization, its interaction with VirB10, and the incorporation of VirB2 and VirB5 into T-pili.

## Introduction

**Figure 1.**
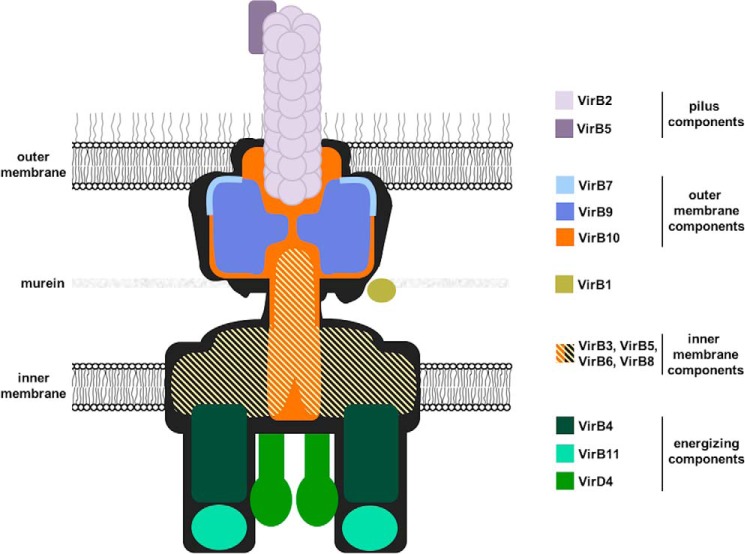
**Model of the type IV secretion system with the known or suspected localization of its 12 components.**

Type IV secretion systems (T4SSs)^3^ mediate the translocation of DNA and protein substrates across the cell envelopes of Gram-negative (Gram−) and Gram-positive (Gram+) bacteria (1, 2). Proteins composing the T4SS are conserved in plant, animal, and human pathogens such as *Agrobacterium tumefaciens*, *Brucella suis*, and *Helicobacter pylori* (3). *A. tumefaciens*, a soil-borne bacterial plant pathogen that is responsible for crown gall disease, uses a T4SS to deliver DNA (a segment of oncogenic DNA from the Ti plasmid) and proteins into plants (4, 5). Its T4SS consists of 12 proteins, VirB1–11 and VirD4, which assemble into a macromolecular complex that spans the inner and the outer membrane (Fig. 1). A combination of X-ray crystallography and cryo-EM solved the structure of the outer part of the T4SS from plasmid pKM101 (6, 7). Transmission EM was used to solve the structure of the VirB3–10 complex from the plasmid R388 T4SS (8), and VirD4 equally localized in this complex (9). This work focuses on the analysis of the interaction between the inner membrane protein VirB6 and VirB10 that is anchored in both bacterial membranes, but currently their exact localization in the T4SS is unknown (8).

VirB6 is a polytopic inner membrane protein that is essential for substrate secretion. It has a periplasmic N terminus and a cytoplasmic C terminus. VirB6 from *A. tumefaciens* potentially comprises five transmembrane (TM) segments and a large periplasmic loop, whereas *in silico* studies predict seven TM domains for VirB6 from *B. suis* and also the presence of a large periplasmic loop (10, 11). Amino acid substitutions in the periplasmic loop reduce the interaction of VirB6 with the T-strand substrate (transferred DNA from the *Agrobacterium* Ti-plasmid coupled to VirD2), and deletions of N-terminal and C-terminal residues abolish substrate transfer to VirB2 and VirB9 (11). VirB6 impacts VirB7 and VirB9 multimerization, and it also stabilizes VirB5; therefore, it is a key component for T-pilus and T4SS assembly (12, 13). VirB6 is believed to act in concert with VirB8 in DNA transfer (11, 14), and the putative periplasmic loop of VirB6 may be the site of interaction (15). We have shown previously that the N terminus of VirB6 from *B. suis* interacts with VirB10 (10), but specific amino acids that form the interaction site have not been identified, and we have no insights into the mechanistic role of this interaction.

VirB10 is another essential component of the T4SS that is anchored both to the inner and the outer membranes, making it a structural scaffold protein (14, 16). It is also considered to be an energy sensor because it undergoes a conformational change induced by the energizing T4SS components VirD4 and VirB11 (16, 17). VirB10 comprises a short N-terminal cytoplasmic region, one TM α-helix, a periplasmic region containing a proline-rich domain and a C-terminal β-barrel domain (18). TraF, a homolog of VirB10 in the conjugative plasmid pKM101, was found to interact in the outer membrane complex with TraN and TraO (homologs of VirB7 and VirB9, respectively) to form a ring-like structure (6, 7, 18).

VirB6 and VirB10 interact via the N-terminal region of VirB6 (10), and deletion of this region impedes DNA substrate transfer to VirB2 and VirB9 (11), suggesting that this interaction is critical for the functionality of the T4SS. The first objective of this study was to map the residues in the N-terminal portion of VirB6 that are essential for its interaction with VirB10. Second, we used *in vivo* studies to evaluate the consequences of amino acid substitutions at the interaction site on T4SS stability and functionality.

## Results

### Analysis with the bacterial two-hybrid system to identify residues of VirB6 involved in the VirB6–VirB10 interaction

**Figure 2.**
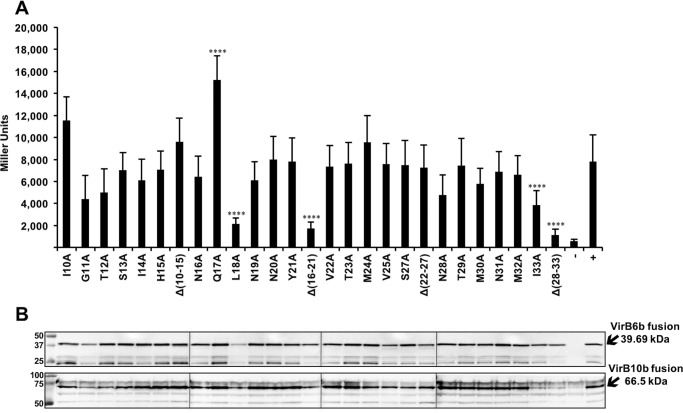
**Results of bacterial two-hybrid analysis to assess the interactions of VirB10b with variants of VirB6b(1–168).** *A*, bacterial two-hybrid assay measuring β-gal activity in the *E. coli* indicator strain BTH101 resulting from the interactions of CyaA fusion proteins to VirB10b and VirB6b(1–168) carrying substitutions of the indicated amino acids by alanine or deletions of blocks of six amino acids. Positive control (+), fusions to VirB10b and VirB6b(1–168) WT and negative control; (−), fusion to VirB10b and a strain not expressing an interaction partner. *B*, analysis of protein levels after SDS-PAGE and Western blotting using anti-CyaA and anti-VirB10b antibodies. Molecular masses of reference proteins are shown on the *left* (in kilodaltons). Values and standard deviations were calculated from five independent experiments. The *p* values were obtained by *t* test, and significant differences were observed between several variants and the WT (****, *p* < 0.0001).

We previously conducted extensive work on *Brucella* T4SS components that, unlike their homologs from *A. tumefaciens*, are easily accessible to biochemical approaches. We used phage display and the bacterial two-hybrid (BTH) assay to identify interaction sites between *Brucella* VirB proteins (10, 15, 19). The information on protein-protein interaction sites is then being applied to test the importance of the interactions between the homologs in T4SS for which more biological readouts are available such as *A. tumefaciens* and pKM101 (20). Following up on our previous work that identified the N terminus as interaction site (10), we determined which residues of VirB6 from *B. suis* (VirB6b) are involved in the interaction with VirB10b. We used the bacterial BTH assay to measure the interaction between a construct expressing the first 168 amino acids of VirB6b and full-length VirB10b, and we changed each of the 24 amino acids (Ile^10^ to Ile^33^) comprising the previously identified VirB10b-binding peptide to alanine (10). Because protein–protein interaction sites typically comprise more than one amino acid, we also deleted four blocks of six amino acids each of this peptide. The BTH results (Fig. 2*A*) show that deletions Δ(16–21) and Δ(28–33) as well as the amino acid substitution L18A strongly reduce the interaction. In contrast, no individual amino acid substitution between residues Asn^28^ and Ile^33^ strongly impacts the interaction. To ensure that we study changes that have a strong impact on the protein–protein interaction, we focused on those that reduce the interaction to less than 50% of the WT level. Western blot analysis showed reduced levels of these VirB6b variants in the cell compared with the WT, and levels of VirB10b were equally reduced (Fig. 2*B*). These results are in accordance with our previous data, suggesting that the fusion proteins mutually stabilize each other and are degraded in the absence of an interaction in the heterologous host *Escherichia coli* (10, 20).

### Effect of changes in the N terminus of VirB6 on the stability of T4SS components

**Figure 3.**
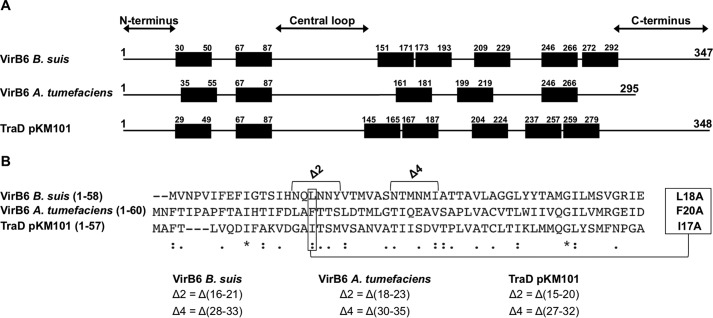
**Sequence alignment between VirB6b, VirB6a, and TraD.** *A*, schematic of the overall topology of VirB6 homologs realized according to the consensus prediction of the membrane protein topology program TOPCONS (38). *B*, CLUSTAL multiple sequence alignment by MUSCLE (3.8), indicating which residues and deletions in VirB6a and TraD correspond to the ones identified with the BTH assay in VirB6b. Sequence similarity is indicated as follows: *asterisks*, identical (single, fully conserved residue); *colons*, conserved substitutions (strongly similar properties); *periods*, semiconserved substitutions (weakly similar properties).

To assess the effects of amino acid substitutions and deletions in the N terminus of VirB6 on the stability and function of the T4SS, we analyzed the effects of complementation with the protein variants *in vivo*. To this effect, we used the *A. tumefaciens* model, for which several different readouts to probe the different steps on T4SS assembly and function are available, as shown in the following. The overall topology of VirB6 homologs is conserved, implying that changes in specific regions likely have similar effects on T4SS assembly and function (Fig. 3*A*). The BTH assays were conducted with the VirB6 homolog from *Brucella*, and we used alignments with the CLUSTAL multiple sequence alignment tool MUSCLE (3.8) to identify the corresponding residues of VirB6 from *A. tumefaciens* (VirB6a) and TraD from the pKM101 T4SS (Fig. 3*B*). This analysis showed that amino acid Leu^18^ and deletions Δ(16–21) and Δ(28–33) from VirB6b correspond to amino acid Phe^20^ and deletions Δ(18–23) and Δ(30–35) in VirB6a and to amino acid Ile^17^ and deletions Δ(15–20) and Δ(27–32) in TraD. We also substituted residues Leu^18^ from VirB6a and Val^21^ from TraD with alanine to serve as controls because we did not expect these changes to have effects based on the results of the BTH analysis.

**Figure 4.**
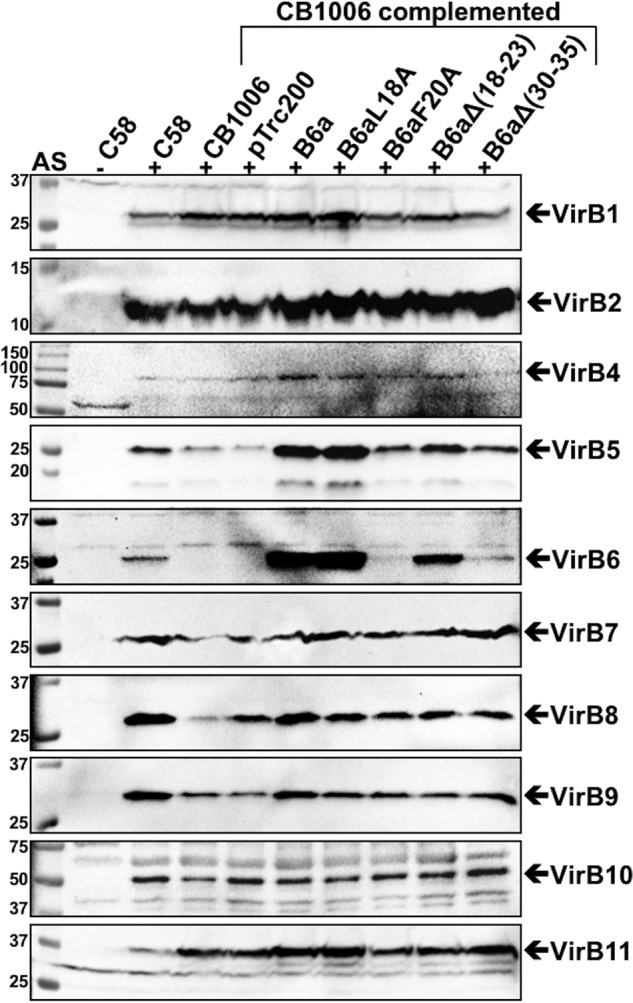
**VirB proteins levels in strains C58 and CB1006 after complementation with VirB6a and its variants.** Shown are results of SDS-PAGE and Western blot analysis with VirB protein–specific antisera of *A. tumefaciens* C58 cultivated without (−*AS*) or with virulence gene induction (+*AS*) and *virB6* deletion (*CB1006*) transformed with empty vector pTrc200- or pTrc200-expressing VirB6a or its variants VirB6aL18A, VirB6aF20A, VirB6aΔ(18–23), and VirB6aΔ(30–35). The signals of the expected molecular masses of VirB proteins are indicated by *arrows*. Molecular masses of reference proteins are shown on the *left* (in kilodaltons). Representative results of five repetitions are shown.

We tested the effects on the complementation of a *virB6* deletion strain of *A. tumefaciens* C58 (CB1006) (13). Expression of VirB6a from pTrc200 leads to overproduction of the protein, even in the absence of the inducer IPTG, and VirB6aL18A and, to a lesser extent, VirB6aΔ(18–23) are also highly expressed (Fig. 4). In contrast, the levels of VirB6aF20A and VirB6aΔ(30–35) are low but reproducibly higher than in the deletion strain CB1006. The levels of VirB5 are strongly reduced in CB1006, and complementation with VirB6a, VirB6aL18A, and VirB6aΔ(18–23) restores the WT level (Fig. 4). Complementation with VirB6aF20A and VirB6aΔ(30–35) increases the level of VirB5 but to levels lower than after complementation with the WT. We did not observe significant effects of deletion or complementation on the levels of any other VirB protein.

### VirB6 and its variants localize in the membrane and show polar localization

**Figure 5.**
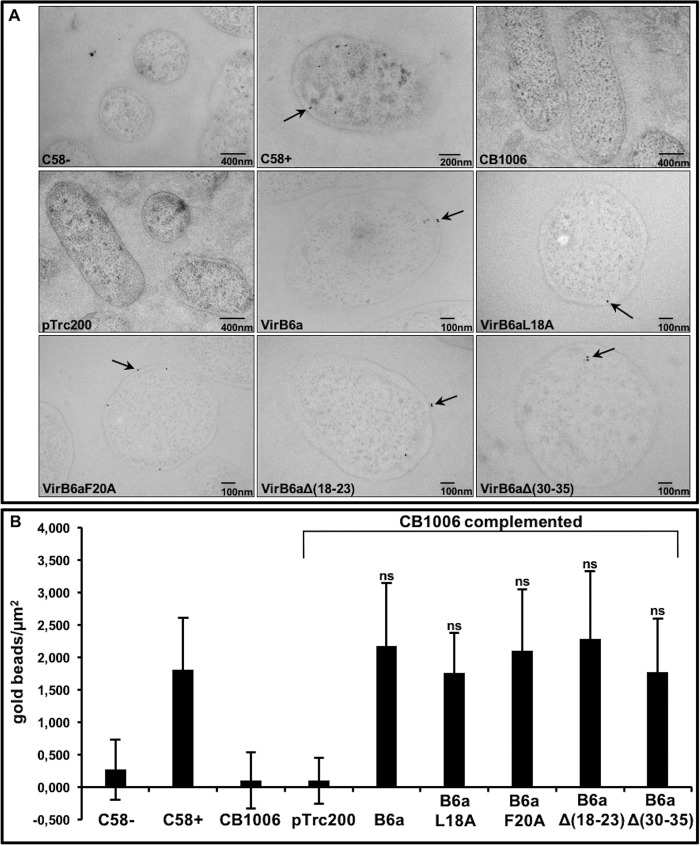
**Immunogold labeling using VirB6-specific antisera to localize Virb6-WT and its variants in *A. tumefaciens*.** *A*, results of immuno-EM analysis with VirB6-specific antisera and colloidal gold–labeled protein A of *A. tumefaciens* C58 cultivated without (−*AS*) or with virulence gene induction (+*AS*) and *virB6* deletion (*CB1006*) transformed with empty vector pTrc200- or pTrc200-expressing VirB6a or its variants VirB6aL18A, VirB6aF20A, VirB6aΔ(18–23), and VirB6aΔ(30–35). Gold beads are indicated by *arrows. B*, graphic representation of the number of gold beads per square micrometer. Values and standard deviations were calculated from three independent experiments. The *p* values were obtained by *t* test, and no significant differences were observed between strain C58 and complemented CB1006. *ns*, *p* ≥ 0.05.

To assess the localization of VirB6a and its variants in *Agrobacterium*, we analyzed the bacteria by EM of thin sections labeled with VirB6-specific primary antibody and colloidal gold–labeled protein A (Fig. 5). The results show that VirB6 is present in the membranes of virulence gene–induced strain C58, it is not detected in CB1006, and it is present in comparable amounts in the membranes of the complemented strains.

**Figure 6.**
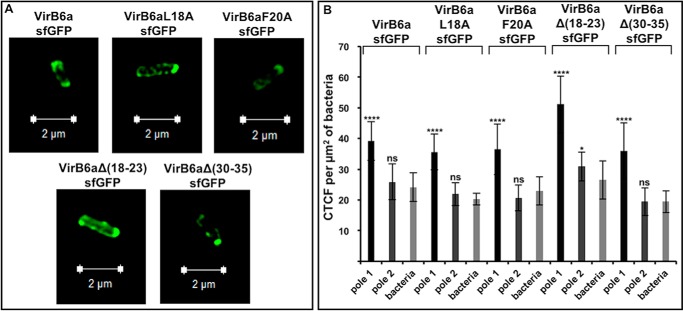
**Structural illumination microscopy of sfGFP-fused VirB6a and its variants.** *A*, C-terminal fusions of VirB6 and of variants VirB6aL18A, VirB6aF20A, VirB6aΔ(18–23), and VirB6aΔ(30–35) to sfGFP were expressed in strain CB1006 and imaged by SIM. *B*, quantification of the fluorescence using the CTCF method, showing preferentially polar localization for each sample (****, *p* < 0.0001). Values and standard deviations were calculated from three independent experiments. The *p* values were obtained by *t* test, for each construction, between the overall bacteria and pole 1 or pole 2. *ns*, *p* > 0.05; *, *p* < 0.05; **, *p* < 0.01; ***, *p* < 0.001, ****, *p* < 0.0001.

Previous work using immunofluorescence microscopy (21, 22) and C-terminal GFP fusion (11) showed that VirB6 localizes to the cell pole. We also analyzed the localization in cells using superfolder GFP (sfGFP) fused to the C terminus of the VirB6a WT and variants. Using structural illumination microscopy (SIM), we demonstrated the preferential localization of VirB6a and of its variants to the cell pole (Fig. 6*A*). Quantification comparing the corrected total cell fluorescence (CTCF) in the bacteria and on each pole demonstrated that there is preferential accumulation at one pole of the cell for the WT and all variants (Fig. 6*B*) and that the overall amounts of fluorescence are similar. Because the results of EM and SIM analysis do not match the differences of the accumulation of VirB6a and some of the variants observed by SDS-PAGE and Western blotting, we next isolated VirB protein complexes by detergent extraction.

### Effect of changes in the N terminus of VirB6 on the stability of T4SS membrane protein complexes

**Figure 7.**
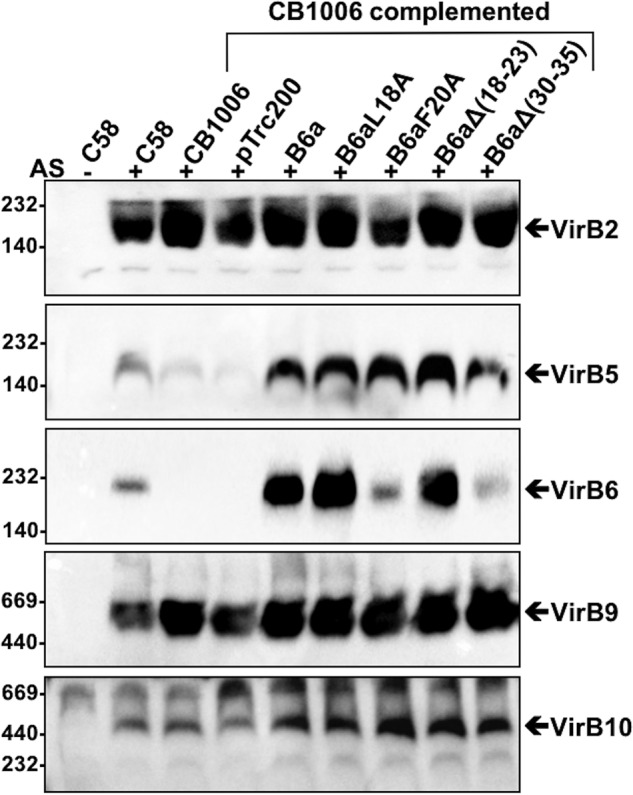
**Native gel electrophoresis to separate detergent-extracted VirB protein complexes.** Membranes were extracted with the detergent DDM from *A. tumefaciens* C58 cultivated without (−*AS*) or with virulence gene induction (+*AS*) and *virB6* deletion (*CB1006*) transformed with empty vector pTrc200- or pTrc200-expressing VirB6a or its variants VirB6aL18A, VirB6aF20A, VirB6aΔ(18–23), and VirB6aΔ(30–35). DDM-solubilized membrane proteins were separated by native PAGE followed by Western blotting with specific antisera and detected in different molecular mass ranges, which are indicated by *arrows*. Molecular masses of reference proteins are shown on the *left* (in kilodaltons). Representative results of four repetitions are shown.

In previous work, we have shown that VirB proteins can be extracted from the membranes in complexes of different sizes that likely reflect functionally important subcomplexes (23). This work suggested that VirB6a is important for the formation of a low-molecular-mass complex of VirB2a and VirB5a that could be a T-pilus preassembly complex (24). We next analyzed whether the variants of VirB6a impact the formation of this complex. Membrane proteins were extracted with dodecyl maltoside (DDM), followed by separation by native gel electrophoreses and Western blotting with specific antisera (Fig. 7). In WT cells, VirB9a and VirB10a were detected in high-molecular-mass complexes of around 500 kDa, whereas VirB2a and VirB5a co-fractionate at around 160 kDa, and VirB6a migrates with a significantly higher molecular mass of about 200 kDa. We did not observe notable differences on the accumulation of VirB2a, VirB9a, and VirB10a, even in the absence of VirB6a. These results suggest that the protein does not impact the stability of these proteins and their complexes in the natural biological context, which is different from the results concerning VirB10 observed in the BTH assay described above. The amounts of VirB6a are similar to those observed after SDS-PAGE of cell lysates showing overexpression of VirB6a as well as of VirB6aL18A and VirB6aΔ(18–23) in the complemented strain. In contrast, the levels of VirB6aF20A and VirB6aΔ(30–35) are lower and similar to VirB6a in the WT strain C58. The levels of VirB5a are similar in all strains expressing VirB6a and its variants. These results suggest that the amino acid substitutions and deletions of VirB6a have no impact on the overall folding of VirB6a, on the formation of the different T4SS subcomplexes, and especially on the stabilization of VirB5a.

### Effect of changes in the N terminus of VirB6 and TraD on T4SS functions

**Figure 8.**
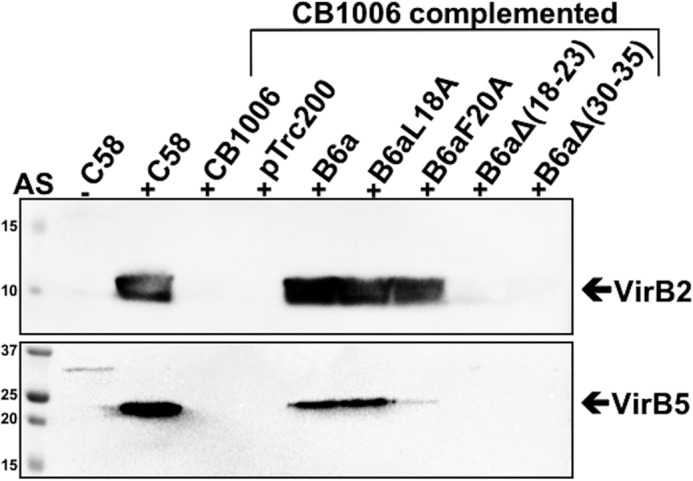
**Analysis of the formation of T-pili.** T-pili were isolated from *A. tumefaciens* C58 cultivated without (−*AS*) or with virulence gene induction (+*AS*) and *virB6* deletion (*CB1006*) transformed with empty vector pTrc200- or pTrc200-expressing VirB6a or its variants VirB6aL18A, VirB6aF20A, VirB6aΔ(18–23), and VirB6aΔ(30–35). High-molecular-mass extracellular structures were isolated from the surface of *A. tumefaciens* by shearing, followed by ultracentrifugation. The T-pilus components VirB2 and VirB5 were detected by SDS-PAGE and Western blotting with specific antisera. Molecular masses of reference proteins are shown on the *left* (in kilodaltons). Representative results of four repetitions are shown.

**Figure 9.**
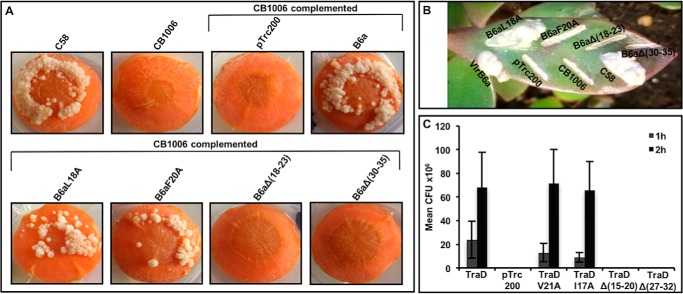
**Virulence and conjugation assays.** *A* and *B*, carrot disc (*A*) and *K. daigremontiana* (*B*) assays showing the formation of tumors after infection with *A. tumefaciens* C58, *virB6* deletion (*CB1006*), and CB1006 transformed with empty vector pTrc200- or pTrc200-expressing VirB6a or its variants VirB6aL18A, VirB6aF20A, VirB6aΔ(18–23), and VirB6aΔ(30–35). Representative results of three repetitions are shown. *C*, conjugation assays between the pKM101-carrying donor strain FM433 and the plasmid-free recipient WL400 were conducted for 1 h or 2 h. Donor cells were pKM101 WT and a *traD* insertion mutant transformed with pTrc200 (empty vector) or complemented with pTrc200-expressing TraD or its variants TraDV21A, TraDI17A, TraDΔ(15–20), and TraD Δ(27–32). Conjugation assays showing the effects of changes on the capacity of TraD and its variants to complement (*CFU*, cfu of the recipient strain). Values and standard deviations were calculated from three independent experiments.

Because the amino acid substitutions and deletions primarily affect the levels of VirB6a in the cells, we conducted T4SS functional assays to determine the importance of the VirB6–VirB10 interaction. First, because VirB6 is important for T-pilus biogenesis and substrate transfer (11) we isolated extracellular high-molecular-mass structures from the cell surface by shearing and ultracentrifugation and tested for the presence of the major T-pilus component VirB2 and the minor component VirB5 (Fig. 8). T-pilus formation is not observed in CB1006, and the defect is complemented by expression of VirB6aL18A and, to a lesser extent, by VirB6aF20A. In contrast, variants VirB6aΔ(18–23) and VirB6aΔ(30–35) do not complement T-pilus formation. Very similar results were observed when the formation of tumors after infection of carrot discs (Fig. 9*A*) and *Kalanchoë daigremontiana* leaves (Fig. 9*B*) was assessed. Complementation with VirB6aΔ(18–23) and VirB6aΔ(30–35) did not restore tumor formation in both assays, whereas VirB6aL18A fully complemented. Expression of VirB6aF20A complements in the carrot disc infection assay (Fig. 9*A*) led to an attenuated phenotype in the *K. daigremontiana* assay (Fig. 9*B*).

To obtain independent evidence for the importance of the N terminus of VirB6, we also tested the functionality of similar changes in its homolog TraD from the pKM101 T4SS that mediates plasmid conjugation. Amino acid substitutions and deletions in regions corresponding to the above changes in VirB6 were introduced into the *traD* gene following sequence alignments, as shown above (Fig. 3). Next, TraD and its variants TraDI17A, TraDV21A, TraDΔ(15–20), and TraDΔ(27–32) were expressed in a pKM101 *traD* insertion strain. Analysis of conjugative plasmid transfer into recipient WL400 shows that TraD variants with single amino acid substitutions (TraDI17A and TraDV21A) fully complement but that the deletion variants TraDΔ(15–20) and TraDΔ(27–32) did not complement the *traD* defect (Fig. 9*C*). The results of functional assays suggest that the interaction between VirB6- and VirB10-like proteins via the N terminus of VirB6 is essential for late stages of T-pilus biogenesis and for T4SS function.

## Discussion

Here we present a detailed analysis of the requirements of amino acids from an interacting peptide of 24 amino acids from the N-terminal region of VirB6 for its interaction with VirB10 and for T4SS function. Although the effects of certain individual amino acid substitutions on the protein–protein interaction are very clear in the BTH assay (more than 70% reduction), analysis of complementation with VirB6aL18A and VirB6aF20A in *A. tumefaciens* shows that the changes do not have very strong effects on overall protein and T4SS function. We observe similar results using equivalent single amino acid substitutions in the pKM101 conjugation system, suggesting that they may be generally applicable for the interaction of VirB6 and VirB10 homologs. The overall sequence similarities between the N termini of VirB6 homologs are low, and we cannot be certain that amino acid changes have identical effects in all homologs. However, the topology of the proteins is actually very similar, suggesting that these regions likely have similar functions.

In contrast, the effects of deletions of blocks of six amino acids that have much stronger effects in the BTH assay (80% to 85% reduction) are much stronger in the *Agrobacterium* and pKM101 complementation assays, showing that the interaction is essential for T4SS function. This distinction is likely because protein–protein interaction sites usually comprise several amino acids. It is therefore difficult to perturb them with single amino acid substitutions. Nevertheless, complementation with the VirB6aF20A variant had an intermediate phenotype, leading to reduced T-pilus formation and tumor formation in the *K. daigremontiana* infection assay. These assays are not quantitative, but the results suggest that amino acid Phe^20^ plays an important role in the protein–protein interaction or in folding of the VirB6 protein. The fact that amino acid substitutions F20A, Δ(30–35), and, to a lesser extent, Δ(18–23) reduce the levels of VirB6a in the cells compared with the overexpressed WT protein suggest that the changes do have an impact on protein stability and that this may be due to folding defects. Nevertheless, they accumulate to levels similar to that in the virulence-induced strain C58, and they stabilize VirB5a, suggesting that the overall impact on their folding is not major.

Using both fluorescence and EM, we did not observe any significant differences in the accumulation of the different VirB6 variants. Also, SIM showed that VirB6a and all variants localize to a single pole of the cells, which is similar to previous observations (21). The results suggest that even the nonfunctional variants arrive at their normal location in the cell in the membrane and at the pole. These observations somewhat contradict the results of SDS-PAGE analysis, and it is possible that this is due to misfolded proteins that may not enter the gels and can therefore not be detected by Western blotting. The results from SDS-PAGE analysis correlate well with those obtained after detergent extraction and native PAGE, showing that VirB6 fractionates in a complex of ∼200 kDa but that the amounts of the F20A and Δ(30–35) variants are reduced compared with the overexpressed WT protein. Nevertheless, the amounts of these proteins in the cell are similar to VirB6 in strain C58, and the levels of the other membrane protein complexes, including VirB5, which is stabilized by VirB6, are similar to WT complementation.

These results suggest that the effects of the amino acid substitutions and deletions on the overall folding and stability of VirB6 and on the stabilization of VirB5 are relatively limited. The observed effects on T-pilus assembly and T4SS functions are therefore due to effects on protein function, suggesting that perturbation of the VirB6–VirB10 interaction prevents T-pilus assembly and T4SS function. Interestingly, after detergent extraction from the membranes, VirB6 and VirB10 fractionate in complexes of two different sizes, and VirB2 and VirB5 co-fractionate in a lower-molecular-mass complex (this work and Ref. 23). Although these complexes are present, and some proteins target themselves to the cell pole (21), T4SS assembly may not be possible because of a lack of interaction between VirB6 and VirB10. This interaction may be a central point for the assembly of the different parts, preventing the recruitment of the VirB2–VirB5 complex to build the pilus (24). Deletion of the *virB6* gene results in a strong decrease in the level of VirB5, and the cells do not assembly T-pili. Strains complemented with VirB6aΔ(18–23) and VirB6aΔ(30–35) are avirulent, and they do not assemble T-pili, but VirB5 and other VirB proteins and their complexes are stable, suggesting that the perturbation of the VirB6–VirB10 interaction explains the phenotype.

**Figure 10.**
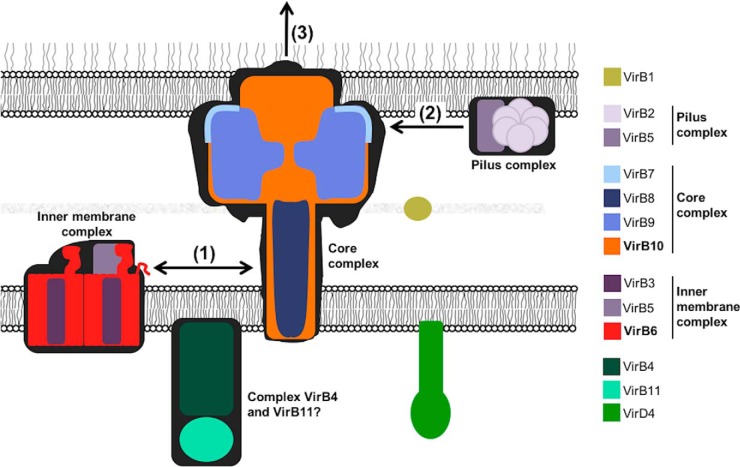
**Model for the role of the VirB6–VirB10 interaction for T4SS assembly.** The interaction between VirB6 and VirB10 (*1*) enables the recruitment of the components of the T-pilus VirB2 and VirB5 (*2*) to the T4SS and is an essential step for pilus biogenesis (*3*).

Deletions of the VirB6 N terminus (Δ1–30, Δ30–60) or C terminus (Δ261–290) abolish substrate transfer to VirB2 and VirB9 (11), and deletion of *virB10* blocks later stages of transfer from VirB6/VirB8 to VirB2/VirB9 (25). These results underline the importance of VirB6 and VirB10 for interactions of VirB2. N- and C-terminal residues of VirB6 are dispensable for substrate trafficking to and across the inner membrane, but the effects of these deletions on T-pilus assembly were not analyzed in previous work (11, 12). The substrate transfer to VirB6 does not depend on pilus formation, and deletions of the genes encoding VirB2 or VirB9 have no effect on its transfer to VirB6 and VirB8 (14, 26). Also, the fact that VirB11 supports pilus production even when substrate transfer is blocked (26) shows that the pilus can be formed even when the translocated substrate does not bind to VirB6. VirB6 may therefore induce pilus formation in a substrate-independent fashion. The pilus assembly signal could be induced when VirB4/VirB11 interact with VirB6, and it is further transmitted via a conformational change of VirB10 to the VirB2–VirB5 complex (Fig. 10) (16, 26). The noncomplementing VirB6 variants stabilize VirB5, but they may not be able to transfer the pilus assembly signal to VirB2-VirB5 because of the absence of interaction with VirB10. This model is consistent with the results of previous studies showing that substrate is not translocated to VirB2/VirB9 when portions of the N terminus (Δ1–30, Δ30–60) of VirB6 are lacking (11).

We already know that VirB6 and VirB8 may link the core T4SS proteins to the pilus assembly complex comprising VirB2 and VirB5 (24). Other studies have shown the importance of VirD4 for the transfer of substrate to VirB11 and VirB4, coordinating substrate transfer to VirB6 (27). Finally, a conformational switch of VirB10 is induced by substrate engagement and contacts between VirD4 and VirB11 (25). To conclude, VirB6 may receive a signal via the VirB4–VirB11 complex, which leads to the recruitment of other components for pilus production (Fig. 10). Simultaneously, VirB10 will undergo a conformational change that may be necessary for recruitment of VirB2/VirB5 and substrate translocation to these proteins. Our results demonstrate that preventing the VirB6–VirB10 interaction leads to defects of the recruitment of VirB2/VirB5 for pilus assembly and of substrate translocation. The absolute levels of VirB6 appear not to be critical for T4SS assembly and function because reduced levels or modest overproduction do not lead to changes of the amounts of T-pili or virulence (11, 12). Future studies of the interaction site of VirB10 with VirB6 should also include VirB8, which is believed to function together with VirB6 in substrate translocation. These interaction sites may be used as targets for inhibitors of T4SS function in bacterial virulence and in plasmid transfer of antimicrobial resistance genes.

## Experimental procedures

### Plasmid construction and DNA modification procedure

The strains and plasmids used are listed in Table S1. To make the *A. tumefaciens* CB1006 strain, an in-frame deletion of *virB6* was introduced into the Ti plasmid of nopaline strain C58, as described previously (10, 28, 29). For complementation experiments, the nopaline Ti plasmid *virB6* gene and the *traD* gene from the IncN plasmid pKM101 were PCR-amplified and cloned in the pTrc200 vector, resulting in pTrcVirB6 and pTrcTraD. To determine the effects of variants in the conjugation assay, strain FM433 carrying a nonpolar transposon insertion in the *traD* gene was transformed with the complementation vector pTrc200 (negative control), pTrc200TraD (positive control), or pTrc200 expressing TraD variants (28, 30).

*E. coli* strain XL-1 Blue was used as a host for cloning and mutagenesis. Standard protocols were used for DNA manipulation using enzymes from New England Biolabs. Cultures for cloning experiments were grown at 37 °C in lysogeny broth (LB). Antibiotics were added for plasmid selection (50 μg/ml spectinomycin, 50 μg/ml streptomycin, 100 μg/ml ampicillin, and 50 μg/ml kanamycin).

### BTH assay

Residues implicated in the interactions between VirB6b and VirB10b were determined *in vivo* using the BTH system (10, 31). The genes encoding VirB6b(1–168) or its variants were fused to the DNA sequence encoding the T18 fragment, and full-length VirB10s was fused to the T25 fragment (32). These fragments correspond to the catalytic domain of *Bordetella pertussis* adenylate cyclase (AC) and are co-expressed in BTH101 AC (*cya*)-deficient cells. The interaction was detected using the functional complementation between the two catalytic AC fragments, leading to cAMP/β-gal production. β-Gal activity was then assessed by using 5-bromo-4-chloro-3-indolyl β-d-galactoside (X-Gal) or *ortho*-nitrophenyl β-d-galactopyranoside (ONPG) as substrate. The transformants were plated onto LB–X-Gal–IPTG (40 μg/ml and 1 mm, respectively) medium, with the two antibiotics (ampicillin (100 μg/ml) and kanamycin (50 μg/ml)) and incubated at 26 °C for 48 h to detect blue or white colonies. Quantification was assayed by growing BTH101 colonies carrying the T18/T25 fusion plasmids in LB with the appropriate antibiotics (ampicillin (100 μg/ml) and kanamycin (50 μg/ml)) overnight at 26 °C with 1 mm IPTG. The β-gal activity was measured from 20-μl aliquots of the cultures mixed with 80 μl of detection buffer (8 mg/ml ONPG, 0.01% SDS, and 50 mm β-mercaptoethanol mixed in Z buffer (0.06 m Na_2_HPO_4_, 0.04 m NaH_2_PO_4_, 0.01 m KCl, and 0.001 m MgSO_4_), pH 7.0). Reaction mixtures were incubated for 2 h at 25 °C, and reactions were stopped with 100 μl of 1 m Na_2_CO_3_. The end products were measured at 420 nm and 550 nm with a 2104 EnVision multilabel plate reader (PerkinElmer Life Sciences). Specific activity was calculated as Miller units = [*A*_420_ − (1.75 × *A*_550_)]/[(t) × *A*_600_ × (volume in milliliters)] × 1000 (*A*_600_, after 12-h incubation; (t), time needed for the color formation). The levels of protein production were assessed using Western blot analyses with anti-CyaA (3D1) mouse antiserum (1:500 dilution) (Santa Cruz Biotechnology) and for VirB6b and VirB10b-specific rabbit antiserum (1:10,000 dilution), which were produced by Biogenes (Berlin, Germany) after injection of rabbits with proteins purified from inclusion bodies.

### SDS-PAGE, native gel, and Western blotting

Cells and protein samples were incubated in Laemmli sample buffer for 10 min at 95 °C, followed by SDS-PAGE. For analysis of VirB6a, samples were lysed with detergent and incubated in Laemmli sample buffer for 15 min at 37 °C, followed by SDS-PAGE. For native gel, proteins solubilized by 2% DDM were mixed with Coomassie Blue G-250 to confer a negative electric charge for electrophoretic separation, followed by native gradient gel electrophoresis (4–16% acrylamide) (23).

Western blotting was performed in a tank blot apparatus, and proteins were transferred to polyvinylidene difluoride membranes. Detection was performed with VirB protein–specific antisera and a chemiluminescence-based solution. *A. tumefaciens* VirB protein–specific antisera were described before (13, 33).

### Bacterial growth and virulence gene induction

For experiments performed with *A. tumefaciens* strain C58 (WT) and its derivatives CB1006 and CB1006 complemented with VirB6 or its variants, cells were grown in YEB medium (10 mg/ml tryptone, 1 mg/ml yeast extract, 5 mg/ml sucrose, and 2 mm MgSO_4_) with spectinomycin (300 μg/ml) and streptomycin (100 μg/ml) for plasmid propagation for 24 h at 26 °C. Following this, cells were inoculated at an *A*_600_ of 0.1 in liquid ABMM+ABSalt 1× (pH 5.5) (ABMM: 10 mg/ml glucose, 3.9 mg/ml MES, 1 mm Na-K-P buffer (0.5 m KH_2_PO_4_ to adjust pH 5.5 by adding Na_2_PO_4_ (0.5 m)); ABSalt: 20 mg/ml NH_4_Cl, 6 g/ml MgSO_4_ × 7H_2_O, 0.2 mg/ml CaCl_2_, 0.05 mg/ml FeSO_4_ × 7H_2_O (pH 5.5)) for 5 h 30 min at 20 °C. After this, 1 ml of bacteria was plated on 30-cm diameter Petri dishes (ABMM+ABSalt solidified with 2% agar) with 200 μm acetosyringone (AS) or without (−AS) for virulence gene induction and further incubated at 20 °C for 4 days. Otherwise, 200 μm AS could be added (or not) directly to the liquid culture and incubated at 20 °C for 20 h for virulence gene induction (13, 34).

### Colloidal gold labeling assays

Bacteria were produced as described above, and cells were sedimented and fixed with 4% paraformaldehyde (Acros Organics, Morris Plains, NJ) and 0.1% glutaraldehyde (Electron Microscopy Sciences, Fort Washington, PA) for 30 min at 4 °C, followed by post-fixation with 1% osmium tetroxide in 0.1 m phosphate buffer (pH 7.2) for 1 h at 25 °C. Between each step, the pellet was washed three times with 0.1 m phosphate buffer. Pellets were then dehydrated using graded alcohol and processed for embedding in LR White resin (London Resin Co., Berkshire, UK). Ultrathin sections of 80- to 100-nm thickness were cut with a diamond knife and collected on formvar-carbon–coated 200-mesh nickel grids. For immunoelectron microscopy, sections were blocked with 1% ovalbumin in 0.01 m PBS and incubated overnight with VirB6a mouse antiserum (1:200 dilution) in PBS. They were then washed using PBS and blocked again before 30-min incubation with 10 nm protein A–coupled gold beads (1:50 dilution, University Medical Center, Utrecht, The Netherlands). Finally, sections were stained with lead citrate for 1 min and observed in a Tecnai 12 transmission electron microscope (FEI Eindhoven, The Netherlands) operated at 120 kV, and images were acquired using a 2 k charge-coupled device (CCD) Advanced Microscopy Techniques (AMT) camera. The gold particles over bacterial profiles were quantified using ImageJ.

### Fluorescence assays

For fluorescence microscopy, 2.5 μl of samples at an *A*_600_ of 4 were spotted on a coverslip and immobilized by 2.5 μl of ProLong Diamond (Life Technologies, Mississauga, ON, Canada). Samples were examined at 30 °C with a ×63/1.4 oil objective under structured illumination microscopy using an Elyra PS1 microscope (Carl Zeiss, Oberkochen, Germany). Proteins linked to sfGFP were excited at 485 nm, and emission around 507 nm was observed using a 14-bit electron-multiplying charge-coupled device (EMCCD) camera. Z-stack volumes were acquired using the SIM and reconstructed using Zen Black edition software. The fluorescence of entire bacteria and of each bacterial pole was quantified using CTCF (35) using ImageJ software.

### Isolation of membranes and detergent extraction

The isolation was conducted following a protocol described previously (23). Briefly, cells were pelleted from 100 ml of ABMM+ABSalt liquid culture. Each pellet was resuspended in the appropriate volume of 50 mm Na-K-P buffer (pH 5.5) to obtain the same amount of bacteria in each sample. 7 ml of each sample was passed through the cell disruptor at 27 kilopounds per square inch (kPsi) twice. Phenylmethylsulfonyl fluoride was added at 0.5 mm after the second round. Cell debris was removed by centrifugation at 3600 × *g* in an SS-34 rotor (Sorvall) for 20 min. Soluble and membrane proteins in the supernatant were then separated by ultracentrifugation for 2 h at 150,000 × *g* in a Sorval T-1270 rotor. Membrane pellets were stored on ice for 12 h. Then, 1 ml of ACA buffer (750 mm 6-amino-caproic acid (ACA) and 50 mm BisTris (pH 7)) was added, and the membranes were suspended by sonication (8 times 10 s; duty, 40%; force 2; Branson sonifier 450). For detergent solubilization, the protein concentration was adjusted to 10 mg/ml, and DDM was added from a 10% stock solution in ACA buffer to give final concentrations of 2%. The samples were mildly shaken for 2 h 40 min at 4 °C, followed by ultracentrifugation for 2 h at 150,000 × *g* in a Sorval T-1270 rotor to separate DDM-soluble and -insoluble proteins. Because of interference from the detergent, protein concentrations in the soluble and insoluble fractions could not be determined at this point.

### Isolation of T-pili

The isolation of T-pili was conducted following a protocol described previously (34). Briefly, cells were grown on ABMM+ABSalt agar in 30-cm-diameter plates (4 plates/sample). Cells from each plate were collected with 5 ml of 50 mm Na-K-P buffer (pH 5.5) and then centrifuged at 12,000 × *g* for 1 h in an SS34 rotor. Cell pellets were suspended in 1 ml of Na-K-P buffer, passed 10 times through a 26-gauge needle to remove surface-associated high-molecular-weight structures and then centrifuged in a microcentrifuge for 1 h 30 min at 14,000 × *g*. Supernatants were subjected to high-speed centrifugation at 230,000 × *g* for 1 h 40 min in a Sorval T-1270 rotor to separate high-molecular-weight structures, like flagella and pili, from other soluble constituents resulting from cell shearing. Pellets were resuspended in 80 μl of Na-K-P buffer.

### Virulence assays

*K. daigremontiana* were injured with a needle and inoculated with 10 μl of *A. tumefaciens* cells (WT and complemented derivatives) grown for 5 h 30 min at 20 °C as described above. Tumor formation was scored after 5–6 weeks.

For the carrot experiments, *A. tumefaciens* was inoculated at 0.1 *A*_600_ in YEB after 24-h cultivation in YEB and placed at 26 °C for 5 h (logarithmic phase culture). During that time, carrots were sterilized with 20% of commercial bleach for 30 min. Under a hood, carrots were rinsed with sterile water and cut into 4- to 5-mm slices. The apical surface was placed on an AB buffer plate (1 mm NaH_2_PO_4_ and K_2_HPO_4_, pH 5.5) and 4 slices/plate were used. Slices were inoculated with 15 μl of 10^9^ cells of *Agrobacterium* harvested and suspended in AB buffer. Slices were placed at 20 °C for 48 h and then transferred to 24 °C. Tumors were monitored 3–4 weeks after inoculation.

### Conjugation assays

*E. coli* FM433 pKM101 donor strains (WT or complemented) and the WL400 recipient strain (chloramphenicol-resistant) were grown in liquid LB medium at 37 °C (with 100 μg/ml ampicillin for pKM101 and 34 μg/ml chloramphenicol for WL400) to an *A*_600_ of 0.5–1, pelleted by centrifugation, and resuspended in an appropriate volume of LB medium to obtain an *A*_600_ of 1 without antibiotics (36, 37). Equal amounts of donor and recipient strains (1 μl of each) were mixed on prewarmed LB agar in 96-well plates and incubated for 1 h or 2 h at 37 °C to enable conjugation. Cells were then washed from the well with 150 μl of liquid LB. To quantify conjugative transfer, dilutions of the conjugation mixture were plated on LB agar plates containing appropriate antibiotics for selection of the pKM101-containing recipient strain WL400 (100 μg/ml ampicillin and 34 μg/ml chloramphenicol).

## Author contributions

C. M. and C. B. conceptualization; C. M., A. F., B. B., and C. B. data curation; C. M., A. F., B. B., A. N., and C. B. formal analysis; C. M., B. B., A. N., and C. B. validation; C. M., A. F., and C. B. investigation; C. M., A. F., and C. B. visualization; C. M., A. F., B. B., and A. N. methodology; C. M. writing-original draft; A. F., A. N., and C. B. writing-review and editing; C. B. supervision; C. B. funding acquisition; C. B. project administration.

## Supplementary Material

Supporting Information

## Footnotes

3 The abbreviations used are:

T4SS: type IV secretion system
TM: transmembrane
BTH: bacterial two-hybrid
IPTG: isopropyl 1-thio-β-d-galactopyranoside
sfGFP: superfolder GFP
SIM: structural illumination microscopy
CTCF: corrected total cell fluorescence
DDM: dodecyl maltoside
LB: lysogeny broth
AC: adenylate cyclase
X-Gal: 5-bromo-4-chloro-3-indolyl β-d-galactoside
ONPG: *ortho*-nitrophenyl β-d-galactopyranoside
AS: acetosyringone
ACA: 6-amino-caproic acid.
