# Supplementary material for: Interaction via the N terminus of the type IV secretion system (T4SS) protein VirB6 with VirB10 is required for VirB2 and VirB5 incorporation into T-pili and for T4SS function
Source: J Biol Chem. 2018 Jul 5;293(35):13415–26. doi: 10.1074/jbc.RA118.002751 (PMC6120205; doi:10.1074/jbc.RA118.002751)
Supplement: Supporting Information [file supp_293_35_13415__index.html]

Interaction via the N terminus of the type IV secretion system (T4SS) protein VirB6 with VirB10 is required for VirB2 and VirB5 incorporation into T-pili and for T4SS function — N-terminal VirB6–VirB10 interaction for pilus assembly — Supporting Information 

# Interaction via the N terminus of the type IV secretion system (T4SS) protein VirB6 with VirB10 is required for VirB2 and VirB5 incorporation into T-pili and for T4SS function

## Supporting Information

- Supplementary Table 1 - Supplementary Table 1
